# A subunit vaccine protects A129 mice from death and joint swelling against lethal Chikungunya virus challenge

**DOI:** 10.3389/fimmu.2026.1937609

**Published:** 2026-10-06

**Authors:** Jiangfan Li, Ting Fang, Liyuan Song, Li Qiang, Yating Hu, Chuanyi Zhao, Haoran Wang, Wenbin Zhao, Guangcheng Fu, Hongyu Yuan, Jianmin Li

**Affiliations:** 1 National Key Laboratory of Advanced Biotechnology, Academy of Military Medical Sciences, Beijing, China; 2 Laboratory of Advanced Biotechnology, ZJU-Hangzhou Global Scientific and Technological Innovation Center, Zhejiang University, Hangzhou, Zhejiang, China

**Keywords:** Chikungunya virus, immunization, protection, safety, subunit vaccine

## Abstract

**Introduction:**

Chikungunya virus (CHIKV) is primarily transmitted to humans through bites of infected *Aedes mosquitoes*, causing febrile illness as well as acute or chronic arthritis. With increasing reports in previously non-endemic regions, it poses an emerging threat to public health and a growing risk to travelers. Despite the recent approval of live-attenuated and virus-like particle-based vaccines, a safe, efficacious, and cost-effective vaccine remains urgently needed. The envelope glycoproteins E1 and E2, which play crucial roles in viral attachment and entry, are the major immunodominant structural proteins and can effectively elicit protective antibodies.

**Methods:**

We engineered mammalian cells to produce the ectodomains of the E1 and E2 glycoproteins and evaluated the protective efficacy of the resulting vaccine formulations *in vivo*.

**Results:**

A two-dose vaccination regimen elicited high levels of antibodies and provided full protection against lethal challenge in A129 mice, preventing both death and joint swelling.

**Discussion:**

These findings demonstrate the potential of this vaccine candidate against CHIKV and warrant its further development.

## Introduction

Chikungunya virus (CHIKV) is a re-emerging mosquito-borne virus belonging to the genus *Alphavirus* within the family *Togaviridae*. Primarily transmitted by *Aedes* mosquitoes, CHIKV is prevalent in tropical and subtropical regions and has spread to more than 100 countries and territories across Asia, Europe, the Americas, and islands in the Indian and Pacific Oceans. Moreover, the adaptation of *Aedes albopictus* to slightly colder environments has broadened the global distribution of CHIKV (1–3). Whether a country had previously reported CHIKV transmission was strongly correlated with the estimated population-weighted presence of *Aedes aegypti* and *Ades albopictus* in the country; consequently, an estimated 2.8 billion people globally are thought to live in areas at risk of CHIKV transmission (4). CHIKV infection can lead to Chikungunya fever (CHIKF) characterized by abrupt onset of fever, headache, and severe polyarthralgia and myalgia. The case-fatality rate for CHIKV disease is approximately 0.1%, with female sex, older age, and comorbidities associated with an increased risk of death (5–7). However, approximately 50% of the infected individuals experience chronic joint pain lasting for months or even years (8–12), causing significant adverse impacts on the economy and society (13, 14).

CHIKV is an enveloped virus approximately 65 nm in diameter, with a single-stranded, positive-sense RNA genome. The genome is approximately 11.8 kb in length, encoding four non-structural proteins (nsP1~nsP4) and five structural proteins (capsid, E3, E2, 6K/TF and E1) (15). The E2 and E1 glycoproteins form a heterodimer, and three E2-E1 heterodimers assemble into a trimeric envelope spike exposed on the virion surface. The E2 glycoprotein interacts with host receptors and facilitates viral binding and attachment (16, 17), while the E1 glycoprotein mediates membrane fusion between the virus and endosomal membrane via pH-dependent endocytosis (18); both processes are essential for viral invasion.

Currently, no specific antiviral therapies exist for CHIKV infection; treatment is primarily directed toward symptomatic relief (19). Non-specific non-salicylate analgesics and non-steroidal anti-inflammatory drugs (NSAIDs) are used for the management of CHIKV infection, but they demonstrate limited clinical efficacy and do not prevent disease transmission (20).

As one of the most effective medical interventions, vaccines play a pivotal role in the prevention and treatment of infectious diseases. Numerous vaccine strategies for CHIKV are currently in development or have been approved, including live-attenuated vaccines (LAVs) (21), inactivated vaccines (22, 23), virus-like particles (VLPs) (24), viral vectored vaccines (25, 26), mRNA vaccines (27, 28), DNA vaccines (29), and subunit vaccines (30, 31). The first licensed vaccine against CHIKV was a live-attenuated vaccine named IXCHIQ (VLA1553). IXCHIQ was approved by the U.S. Food and Drug Administration (FDA) in November 2023. IXCHIQ is administered as a single intramuscular dose to individuals aged 18 years and older at increased risk of CHIKV exposure. It was subsequently approved by the European Medicines Agency (EMA) in June 2024 following a stringent assessment (32). Notably, the licensure was based on a correlate of protection rather than traditional phase 3 trials, owing to the unpredictable nature of CHIKV epidemiology (4). However, the FDA suspended its marketing on August 2025 due to severe adverse events reported during its use (33). In February 2025, the FDA approved a virus-like particle vaccine named VIMKUNYA for individuals aged 12 years and older. VIMKUNYA demonstrated a favorable safety profile, with most adverse events reported as mild and self-limiting, and robust immunogenicity, achieving a seroresponse rate of 97.8% in a phase 3 trials (34). Nevertheless, the production of VLP is technically challenging, potentially limiting scale-up and increasing costs (35). As CHIKV predominantly affects low- and middle-income countries (36), a safe and affordable vaccine is urgently needed.

In this study, we developed an effective CHIKV subunit vaccine based on the ectodomain of the envelope proteins (16). We constructed and produced the recombinant subunit proteins and evaluated their immunogenicity and protection efficacy against attenuated CHIKV in mice. The subunit vaccine induced robust humoral immune responses, completely protecting the mice from death and joint swelling while significantly reducing viral load. These findings indicate that the subunit protein represents a promising alternative vaccine candidate against CHIKV.

## Materials and methods

### Animals and ethics statement

All animal experiments were conducted in strict compliance with the ethical guidelines outlined in the National Regulations for the Administration of Laboratory Animals and received approval from the Institutional Animal Care and Use Committee at the Laboratory Animal Centre of the Beijing Institute of Biotechnology (approval number IACUC-SWGCYJS-2025-035).

BALB/c mice aged 6–8 weeks, used for immunogenicity assessment, were purchased from SPF (Beijing) biotechnology co., LTD (Beijing, China). A129 mice used for the assessment of protection efficacy were purchased from Shanghai Model Organisms Center, Inc. (Shanghai, China). For challenge studies, mice were housed in micro-isolator cages on a ventilated rodent rack in an animal biosafety level 2 (ABSL-2) facility.

### Cells and viruses

The human embryonic kidney cell line (HEK293T, ATCC L-11268), baby hamster kidney fibroblast cell line (BHK-21, ATCC CRL-8544) were cultured at 37°C and 5% CO_2_ in Dulbecco’s Modified Eagle’s Medium (DMEM) (Gibco C11995500BT) supplemented with 10% fetal bovine serum (FBS), 100 μg/mL streptomycin, and 100 IU/mL penicillin. Chinese hamster ovary (CHO) cells were cultured at 120 rpm, 36.5°C, and 5% CO_2_ in Dynamis Medium supplemented with 1% hypoxanthine and thymidine (HT), 1% anti-clumping agent (37), and 2.5% Cell Boost 5 (cytiva).

A live-attenuated strain of CHIKV (38) was propagated in BHK-21 cells grown in RPMI 1640 medium supplemented with 2% FBS at 37°C as described previously (39).

### Protein production

The vaccine construct was based on the CHIKV strain 37997 (GenBank EU224270). Sequences encoding signal peptide (MTRLTVLALLAGLLASSRA), E3, E2 ectodomain (aa 1-341), (GGGGS)_4_ linker, and E1 ectodomain (aa 1-419) were codon-optimized and inserted in to pNRC expression vector. CHO cells were transfected with the recombinant plasmid and screened using L-methionine sulfoximine (MSX).

For protein purification, the cell culture supernatant was collected and clarified via filtration. The sample was then purified by ion-exchange chromatography using a Capto S Impact column (cytiva), followed by ultrafiltration to exchange the buffer to 50 mM sodium acetate-acetic acid (NaAC-HAC). To obtain protein of higher purity, an additional gel filtration chromatography step was performed using a Chromdex 200 PG column (cytiva).

Recombinant E proteins from the three additional representative strains (AF15561, LR2006, and S27) were generated by transient transfection and purified using the same methods as described above.

### Western blot analysis

The composition of the purified subunit protein was analyzed by Western blot. Purified protein samples were mixed with 5× reducing sample buffer and heated at 100°C for 10 min. Prepared samples, along with a pre-stained protein marker (Thermo Scientific, 26617), were resolved on a 4-12% gradient SDS-PAGE gel (GenScript) and subsequently transferred to nitrocellulose membranes. The membranes were blocked with 5% (w/v) skim milk in PBST (phosphate-buffered saline containing 0.05% Tween 20) for 2 h at room temperature with gentle agitation. They were then incubated separately for 1 h at room temperature with one of the following primary antibodies: anti-CHIKV E3 polyclonal antibody (GeneTex), anti-CHIKV E2 monoclonal antibody (CHK-265) (40), or anti-CHIKV E1 monoclonal antibody (N046) (39). After three washes with PBST (10 min each), the membranes were incubated for 1 h with horseradish peroxidase-conjugated goat anti-rabbit IgG or goat anti-human IgG (Abcam), diluted 1:10,000 (v/v) in blocking buffer. Following three additional washes with PBST, immunoreactive bands were detected using a chemiluminescent substrate (Millipore) and visualized with an iBright 1500 imaging system (Thermo Scientific).

### Enzyme-linked immunosorbent assay (ELISA)

ELISA 96-well plates (Corning) were coated with 1.0 μg/mL of the subunit protein overnight at 4°C. With the exception of the cross-reactivity evaluation, all other ELISAs were performed using the subunit protein of the strain 37997. Plates were washed with PBST (PBS + 0.05% Tween 20) and blocked with blocking buffer (PBST + 3% BSA) for 1 h at 37°C. Plates were washed with PBST, and serially diluted sera were added and incubated 45 min at 37°C. After washing, horseradish peroxidase (HRP)-conjugated anti-mouse antibodies were added and incubated 30 min at 37°C. After washing, 3,3’,5,5’-tetramethylbenzidine (TMB) was added and incubated in the dark for 6 min at room temperature. The reaction was stopped by adding 2 M H_2_SO_4_. Absorbance values were measured at 450 nm with a reference wavelength of 630 nm. The endpoint titer was defined as the highest reciprocal dilution of serum to give an absorbance value greater than 2.1-fold that of the naive serum. Antibody titer below the limit of detection was assigned as half the limit of detection.

### Enzyme-linked immunospot assay (ELISpot)

CHIKV-specific cellular immune responses in mice were assessed using a mouse IFN-γ ELISpot kit (MabTech). In brief, ELISpot plates were coated overnight at 4°C with 5 μg/mL anti-mouse IFN-γ capture antibody. After washing five times with sterile PBS, plates were blocked with complete RPMI 1640 medium for 2 h at room temperature. After blocking, 100 μL of splenocyte suspension (2 × 10^6^ cells/mL) were added to each well and stimulated with overlapping peptide pools (2 μg/mL). The plates were incubated for 24 h at 37°C under 5% CO_2_. Following five washes with PBS, biotinylated anti-mouse IFN-γ detection antibody was added, and the plates were incubated at room temperature for 1 h. After five additional washes, streptavidin-HRP was added, and the plates were incubated at room temperature for 1 h. Following a final wash, TMB substrate was applied to develop spots. Finally, the plates were washed thoroughly with distilled water to terminate color development and allowed to dry in the dark. Spots were recorded utilizing an ELISpot reader (AID GmbH).

### Pseudotyped virus neutralization assays

The serum to be tested was heat-inactivated at 56°C for 30 min. The inactivated serum samples were serially diluted and incubated with 250 TCID_50_ of CHIKV pseudotyped virus for 1 h at 37°C. HEK293T cells were then added and incubated for 48 h. Subsequently, cells were lysed, and relative light unit (RLU) were measured using the GloMax Navigator (Promega). The 50% inhibitory dilution (ID_50_) is defined as the neutralizing antibody titer of the serum.

### Immunogenicity studies in mice

BALB/c mice (6–8 weeks old, *n* = 5 per group, 3 females and 2 males) were immunized by intramuscular injection on day 0 and 21. David et al. demonstrated that two doses of 1 μg of p62-E1 protein (produced in insect cells) adjuvanted with AS03 or Matrix-M elicited high levels of CHIKV-specific IgG in mice (41). Manisha et al. reported that three doses of 10 μg of E2 protein (produced in a bacterial expression system) adjuvanted with aluminum induced moderate IgG responses (42). Therefore, a 5 μg dose was chosen to ensure adequate immunogenicity while minimizing the required antigen amount. The total injected volume was 100 μL, administered as 50 μL per thigh. The PBS-immunized group served as a control. Mice in other groups were immunized with 5 μg of subunit protein, either alone or formulated with 75 μg of aluminum hydroxide (Biodragon), or with 50 μL of BFA01, BFA03, BFA04 (Recbio Tech), or Ribi (Sigma) per mouse. Blood samples were collected on day -1, 14, and 35 for antibody detection.

### *In vivo* protection studies in mice

The protective efficacy of the vaccines was assessed using an A129 (IFNAR1-knockout) mouse model. Mice were immunized intramuscularly with PBS or adjuvanted vaccines on day 0 for single-dose administration, or on day 0 and day 21 for two-dose administration. Two weeks after the final vaccination, mice were bled for antibody detection and challenged the next day via footpad injection with 2 × 10^4^ TCID_50_ of the attenuated CHIKV (strain LR2006). The mice were observed daily for mortality, body weight, and ipsilateral/contralateral joint swelling for up to 14 days post-infection. On day 3 post-infection, two or three mice from each group were euthanized for viral load determination. Euthanasia was performed using CO_2_ delivered at a flow rate of 2.5 L/min (approximately 25% of the 10-L chamber volume per minute) to achieve gradual filling and minimize distress, followed by cervical dislocation.

For passive transfer experiments, sera from BALB/c mice collected 9 weeks after the aforementioned booster immunization were pooled, and total IgG was purified by Protein G affinity chromatography. Sera were obtained from the PBS control group and the BFA01-adjuvanted vaccine group. Purified antibodies or PBS were administered intravenously to naive A129 mice. Each mouse received a total volume of 100 μL containing either 100 μL PBS or 100 μg purified IgG. Twenty-four hours later, mice were challenged via footpad inoculation with 2 × 10^4^ TCID_50_ of attenuated CHIKV. Survival rates, body weights, and footpad thicknesses were monitored. On day 3 post-infection, two mice from each group were euthanized for viral load determination.

### Viral load determination using RT-qPCR

RNA was isolated from tissues using the RNeasy Mini Kit (Qiagen) according to the manufacturer’s instructions. Reverse transcription of cDNA was performed using HiScript III RT SuperMix (Vazyme), followed by RT-qPCR using 2 × Taq Pro Universal SYBR qPCR Master Mix (Vazyme) with the following primer pair targeting the region spanning the C-terminus of E2 protein and the N-terminus of the 6K/TF protein (nucleotides 9756 to 9866):

Forward, 5′-AGCTACCGTCCCTTTCCTGCTTA-3′;

Reverse, 5′-CAAAACAAAGGTTGCTGCTCGTT-3′.

## Results

### Design and production of recombinant subunit protein

The recombinant CHIKV subunit construct contains a signal peptide, E3, the E2 ectodomain (amino acids 1-341), a (G_4_S)_4_ linker, and the E1 ectodomain (amino acids 1-419) (**Figure 1A**). To enhance protein expression and secretion, a signal peptide (SP) was added to the N-terminus. No purification tag was included. The construct was transfected into Chinese hamster ovary (CHO) cells, and the subunit protein was successfully expressed (**Figure 1B**). Stable cell lines were subsequently established, and the clone with the highest expression level was selected. The expression level reached 0.5 g/L. The supernatant containing the subunit protein was collected, followed by ion-exchange chromatography (**Figure 1C**), ultrafiltration for buffer exchange, and further gel filtration chromatography. Subsequently, the subunit protein was obtained at high purity (**Figure 1D**) and used for further study. Western blot analysis confirmed the presence of E3, E2, and E1 (**Figure 1E**).

**Figure 1:**
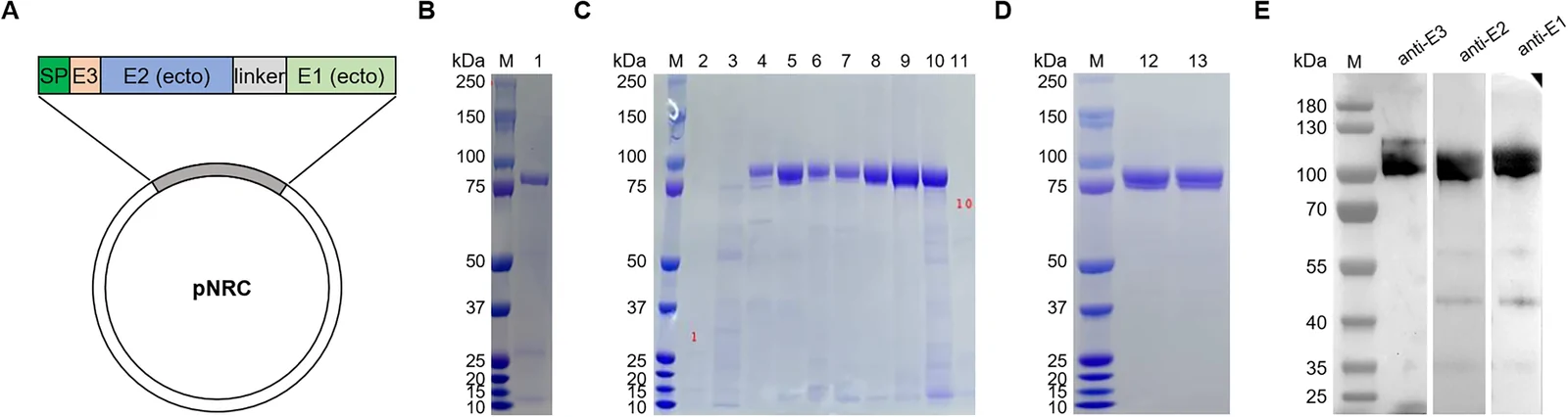
Design, expression and purification of the subunit protein. **(A)** Schematic of the CHIKV subunit vaccine construct. SP: signal peptide; ecto: ectodomain. **(B)** Verification of the subunit protein expression via transient transfection. **(C)** Samples collected following ion-exchange chromatography. **(D)** Purity identification after gel filtration chromatography. M lane: marker; lane 2-11: eluted samples. **(E)** Western blot analysis of purified subunit protein.

### Vaccine immunogenicity in BALB/c mice

To evaluate vaccine immunogenicity, groups of BALB/c mice (*n* = 5) were immunized with two doses of 5 μg subunit protein, 21 days apart (**Figure 2A**). To determine a suitable adjuvant for the vaccine, we selected several adjuvants, including the widely used aluminum hydroxide and some novel adjuvants, BFA01 (containing MPL, QS-21 etc.), BFA03 (containing squalane, tocopherol etc.), BFA04(containing MPL, aluminum etc.), and Ribi (containing MPL, TDM etc.). PBS alone was administered as a control. As expected, PBS alone failed to elicit any antigen-specific antibodies two weeks after either the prime or boost immunization (**Figures 2B–E**). Without adjuvant, the subunit protein alone elicited virtually no antigen-specific antibodies after the prime (**Figure 2B**) and only very low antibody levels after the boost (**Figures 2C–E**). After the boost, the aluminum-adjuvanted vaccine elicited high levels of IgG and IgG1 (**Figures 2D, E**), but a relatively low level of IgG2a (**Figure 2E**). In comparison, all vaccines adjuvanted with the novel adjuvants elicited significantly higher levels of IgG2a (**Figure 2E**). Notably, the BFA01-adjuvanted vaccine elicited the highest level of IgG2a. Together, these results suggest that the aluminum hydroxide induced a Th2-biased immune response, whereas the novel adjuvants elicited a relatively balanced Th1/Th2 immune response. A pseudotyped virus neutralization assay was performed to evaluate the neutralizing activity of the sera. The results showed that all the adjuvanted vaccines elicited moderate neutralizing antibody titers (**Figure 2F**).

**Figure 2:**
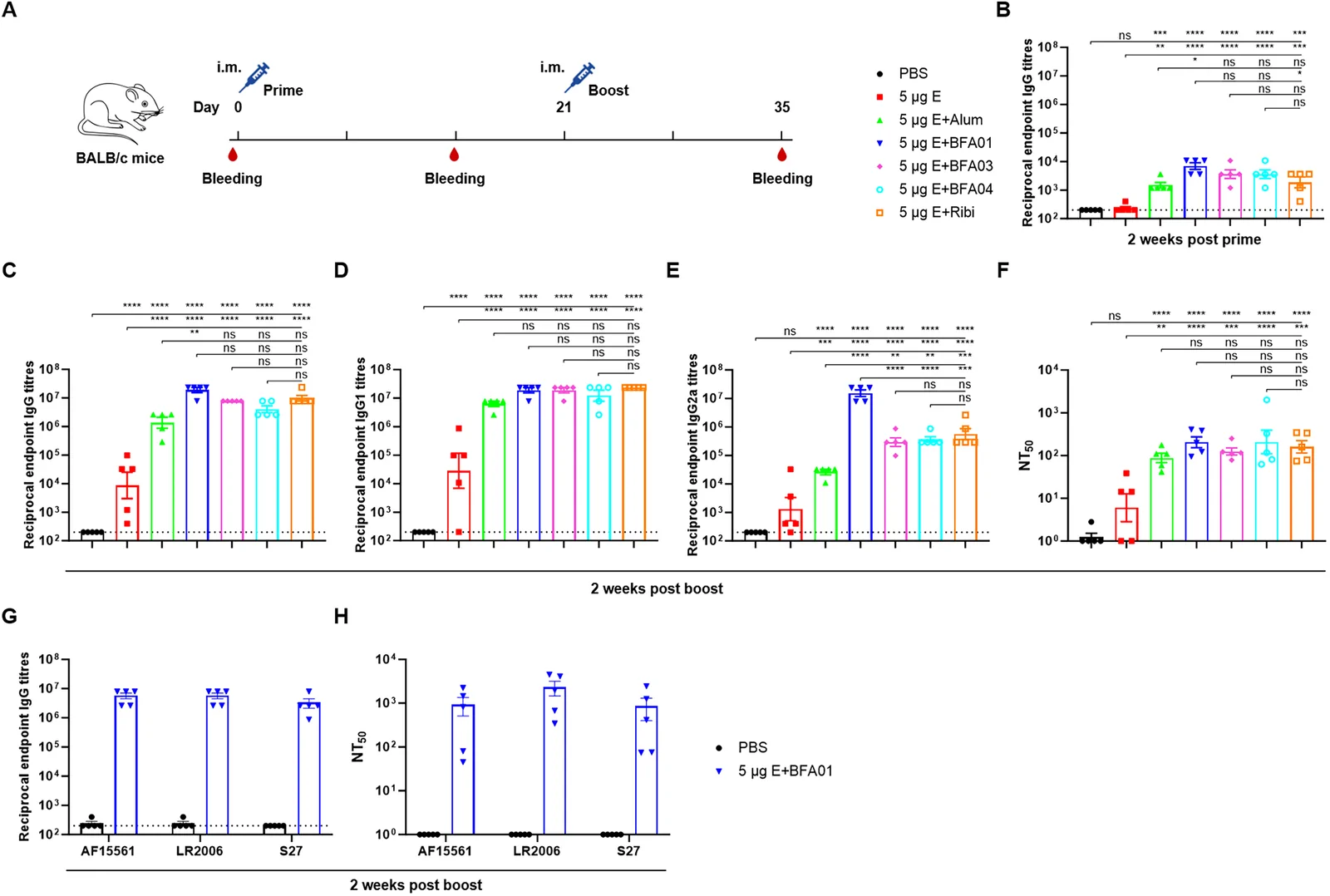
Immunogenicity of the vaccines in mice. **(A)** Experimental schedule. **(B)** Detection of CHIKV-specific IgG post prime by endpoint ELISA. Detection of CHIKV-specific IgG **(C)**, IgG1 **(D)**, IgG2a **(E)** by endpoint ELISA and neutralizing antibodies post boost by pseudotyped virus neutralization assay. Panels B-F show the evaluations for strain 37997. **(G)** Detection of cross-reactive CHIKV-specific IgG post boost by endpoint ELISA. **(H)** Detection of cross-neutralizing antibodies post boost by pseudotyped virus neutralization assay. Significance was determined by one-way analysis of variance with Tukey’s multiple-comparison test; *p<0.05, **p<0.01, ***p<0.001, ****p<0.0001; ns, not significant; NT_50_, half neutralization titer. Data are represented as mean ± SEM. *n* = 5/group (three females, two males).

Cellular immune responses were preliminarily assessed by IFN-γ ELISpot using splenocytes collected 7 days post-boost. Spot-forming units were low across all groups, and no clear antigen-specific response above background was observed (**Supplementary Figure S1**).

To evaluate the broad-spectrum protective potential of the vaccine, we first assessed the cross-reactivity of serum from immunized mice against envelope proteins from other lineages. Using the same expression strategy, we produced and purified recombinant E proteins from three additional representative strains: the Asian genotype (AF15561), the East/Central/South African (ECSA) genotype (S27), and the Indian Ocean lineage (IOL) (LR2006 OPY-1). ELISA revealed high specific IgG titers in serum from the BFA01-adjuvanted vaccine group against all three envelope proteins (**Figure 2G**). We then evaluated the neutralizing activity of the serum against pseudotyped virus corresponding to these three strains. Mouse immune serum potently neutralized all three pseudotyped viruses (**Figure 2H**), providing preliminary functional evidence of cross-lineage neutralizing activity.

### Vaccine protection against attenuated CHIKV in A129 mice

To assess vaccine protective efficacy, groups of A129 (IFNAR1-knockout) mice were administered two doses of PBS (control) or different vaccine formulations (**Figure 3A**). Two weeks after the last immunization, mice were bled for antibody detection. The results showed that aluminum-adjuvanted vaccine elicited high levels of IgG and IgG1 and moderate levels of IgG2a after the boost (**Figures 3B–D**), consistent with the results observed in BALB/c mice. One study indicated that CHIKV-neutralizing antibodies are mainly IgG3 in humans (43). Thus, we assessed the levels of IgG3. The results showed that BFA01-adjuvanted vaccine elicited a relatively high level of IgG3, whereas the aluminum-adjuvanted vaccine only elicited a modest level of IgG3 (**Figure 3E**).

**Figure 3:**
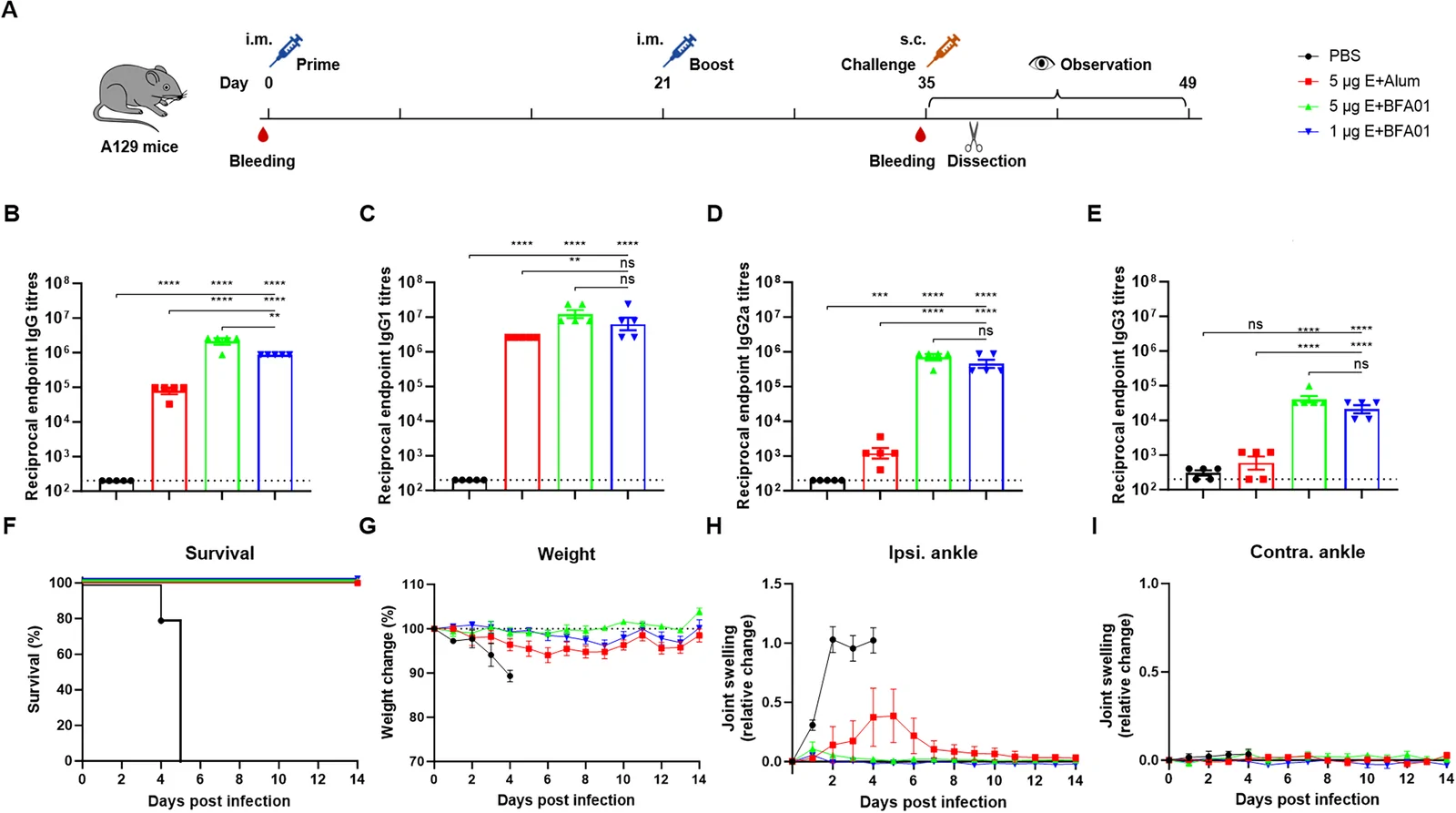
Protection efficacy of two-dose vaccination. **(A)** Experimental schedule. Detection of IgG **(B)**, IgG1 **(C)**, IgG2a **(D)**, and IgG3 **(E)** before challenge by endpoint ELISA. Significance was determined by one-way analysis of variance with Tukey’s multiple-comparison test; *p<0.05, **p<0.01, ***p<0.001, ****p<0.0001; ns, not significant. Survival **(F)**, weight change **(G)**, ipsilateral joint swelling **(H)**, and contralateral joint swelling **(I)** were monitored 14 days post-infection. Data are represented as mean ± SEM. *n* = 5/group (five females).

Notably, compared with 5 μg subunit protein adjuvanted with aluminum, 1 μg subunit protein adjuvanted with BFA01 elicited comparable or even higher levels of antibodies (**Figures 3B–E**), suggesting that BFA01 may allow for antigen dose sparing.

The mice were then challenged with the attenuated CHIKV one day after blood collection and observed for two weeks. As expected, all mice in the control group died within 5 days post-infection. Immunization with any of the three vaccines resulted in a 100% survival (**Figure 3F**) and significantly prevented weight loss (**Figure 3G**). In addition, in the control group, CHIKV inoculation in the footpad resulted in joint swelling in the ipsilateral ankle. Two mice in the aluminum-adjuvanted group showed moderate joint swelling in the ipsilateral but recovered quickly within several days (**Figure 3H**). Mice in the BFA01-adjuvanted group showed no obvious joint swelling in the ipsilateral ankle. No obvious joint swelling was observed in the contralateral ankle in any group (**Figure 3I**).

On day 3 post-infection, two or three mice from each group were euthanized for viral load determination in the liver, spleen, lung, kidney, brain, muscle, and ankle by RT-qPCR. Euthanasia was performed using CO_2_ delivered at a flow rate of 2.5 L/min (approximately 25% of the 10-L chamber volume per minute) to achieve gradual filling and minimize distress, followed by cervical dislocation. The results showed that all three vaccine formulations significantly reduced viral load in most tissues compared with PBS (**Figure 4**). Differences among the three groups were negligible. In addition, none of the mice showed obvious pathological change in the above tissues on day 3 post-challenge (**Supplementary Figure S2**), even in the control group.

**Figure 4:**
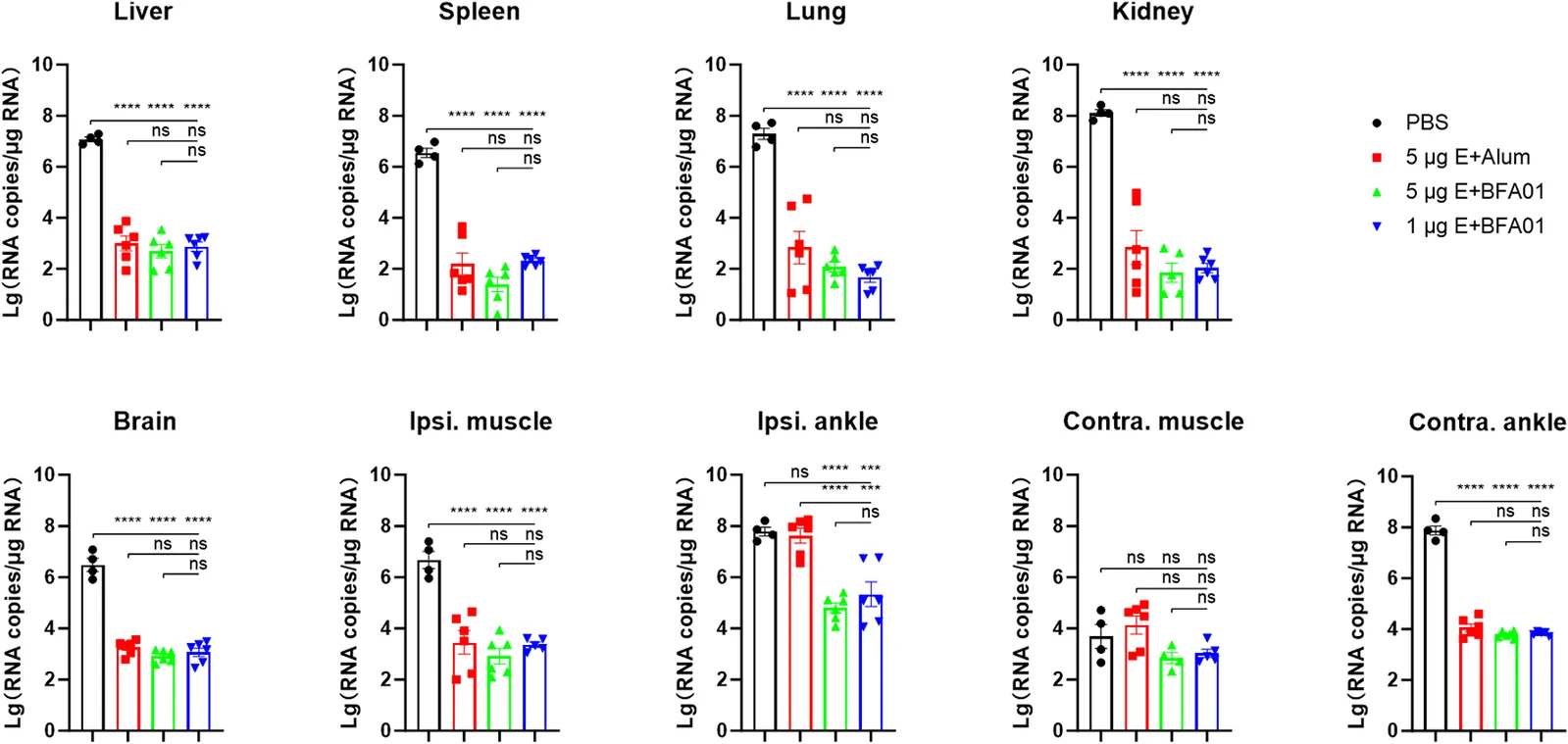
Viral loads. Virological assessment by RT-qPCR at 3 dpi in the liver, spleen, lung, kidney, brain, ipsilateral ankle/muscle, and contralateral ankle/muscle. Significance was determined by one-way analysis of variance with Tukey’s multiple-comparison test; *p<0.05, **p<0.01, ***p<0.001, ****p<0.0001; ns, not significant. Data are represented as mean ± SEM. *n* = 2 (PBS group, male) and *n* = 3/group (other groups, male).

We next sought to determine whether a single dose of immunization could afford some degree of protection. A129 mice received a single administration of the same preparations as described above. Mice were bled two weeks after immunization and challenged with the attenuated CHIKV the following day (**Figure 5A**). The results showed that 5 μg subunit protein adjuvanted with BFA01 elicited the highest antibody levels (**Figures 5B–E**). After challenge, the BFA01-adjuvanted vaccines conferred 100% survival, whereas the aluminum-adjuvanted vaccine conferred 80% survival (4/5) (**Figure 5F**). All vaccines alleviated the weight loss to varying degrees (**Figure 5G**), but only mice vaccinated with 5 μg BFA01-adjuvanted subunit protein nearly recovered to their pre-challenge weight levels by the end of the observation period. Unlike the two-dose regimen, all mice showed varying degrees of joint swelling in the ipsilateral ankle (**Figure 5H**). In the group vaccinated with 5 μg BFA01-adjuvanted subunit protein, the ipsilateral joint swelling subsided within approximately one week, whereas the contralateral joint remained free of obvious swelling (**Figure 5I**).

**Figure 5:**
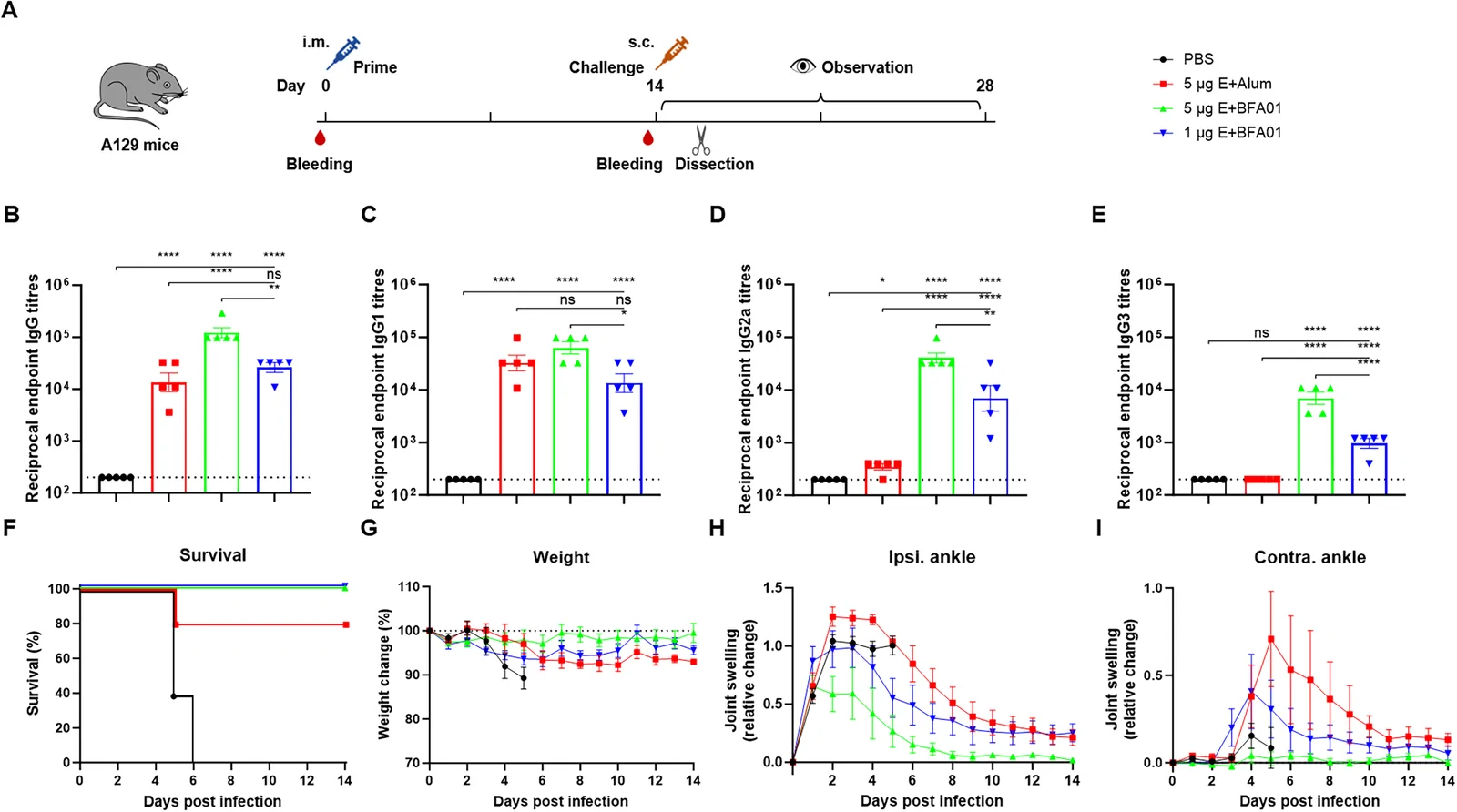
Protection efficacy of single-dose vaccination. **(A)** Experimental schedule. Detection of IgG **(B)**, IgG1 **(C)**, IgG2a **(D)**, and IgG3 **(E)** before challenge. Significance was determined by one-way analysis of variance with Tukey’s multiple-comparison test; *p<0.05, **p<0.01, ***p<0.001, ****p<0.0001; ns, not significant. Survival **(F)**, weight change **(G)**, ipsilateral joint swelling **(H)**, and contralateral joint swelling **(I)** were monitored 14 days after infection. Data are represented as mean ± SEM. *n* = 5/group (five females).

Similarly, the viral loads in the tissues were determined on day 3 post-infection. The results showed that 5 μg BFA01-adjuvanted subunit protein significantly reduced viral loads in most tissues, whereas the other two formulations only moderately reduced viral loads in some tissues (**Figure 6**). These results were consistent with the clinical performance observed in mice (**Figures 5F–I**).

**Figure 6:**
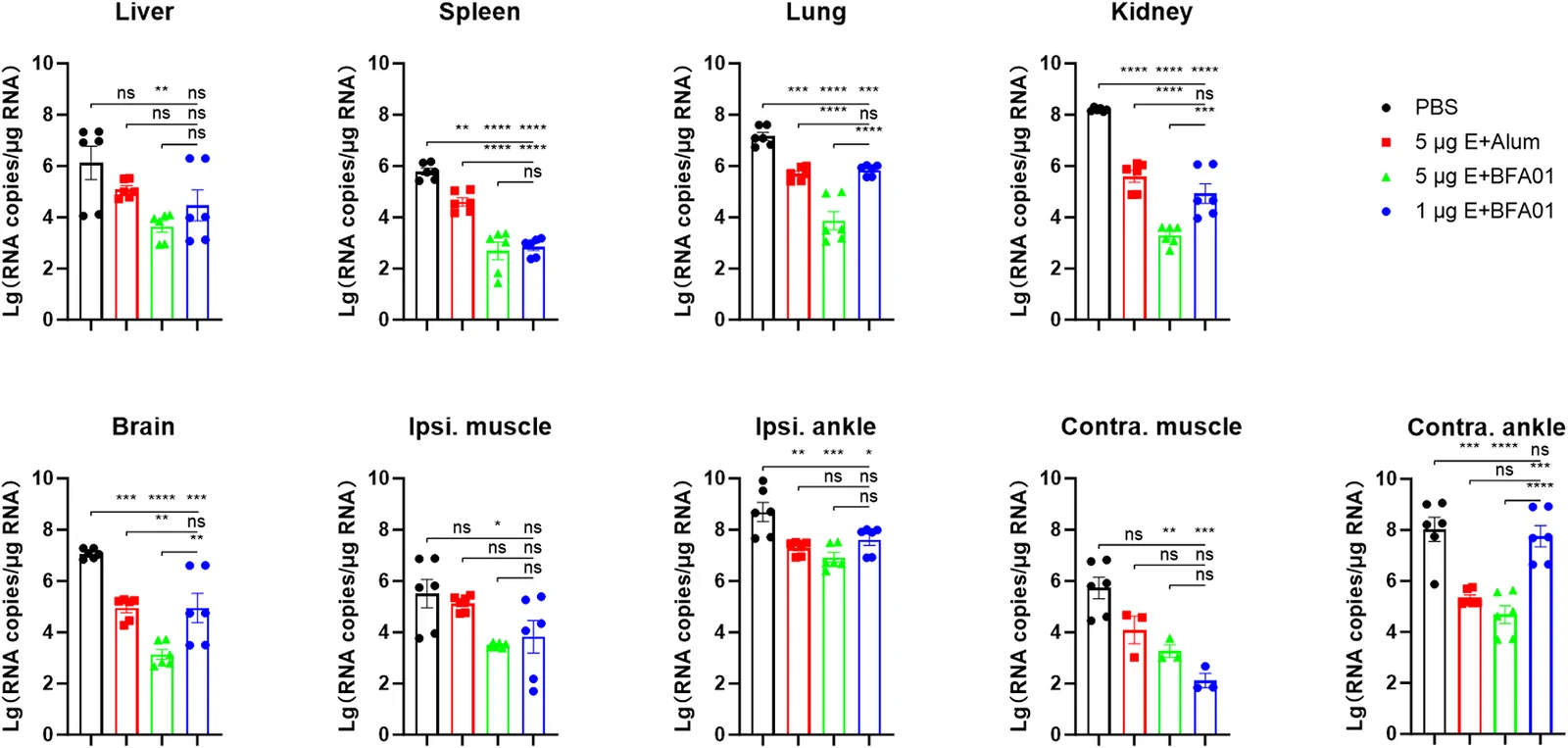
Viral loads. Virological assessment by RT-qPCR at 3 dpi in the liver, spleen, lung, kidney, brain, ipsilateral ankle/muscle, and contralateral ankle/muscle. Significance was determined by one-way analysis of variance with Tukey’s multiple-comparison test; *p<0.05, **p<0.01, ***p<0.001, ****p<0.0001; ns, not significant. Data are represented as mean ± SEM. *n* = 3/group (three males).

### Protection of purified IgG via passive transfer

To demonstrate that the vaccine-induced protection observed *in vivo* was mediated, at least in part, by serum antibodies, a passive transfer experiment was performed. IgG was purified from sera of mice immunized with PBS or the BFA01-adjuvanted vaccine and transferred into naive A129 mice. PBS transfer served as the control. Twenty-four hours later, the mice were challenged with the attenuated CHIKV. By day 6 post-infection, all mice receiving PBS or IgG from PBS-immunized mice had succumbed, whereas all mice receiving IgG from mice immunized with BFA01-adjuvanted vaccine survived (**Figure 7A**). Body weight declined rapidly in all groups by day 3 post-infection (**Figure 7B**), after which surviving mice gradually recovered from day 7 onward. However, all surviving mice developed marked joint swelling in both ipsilateral and contralateral ankles (**Figures 7C, D**). After day 6 post-infection, surviving mice gradually regained body weight and joint swelling subsided.

**Figure 7:**
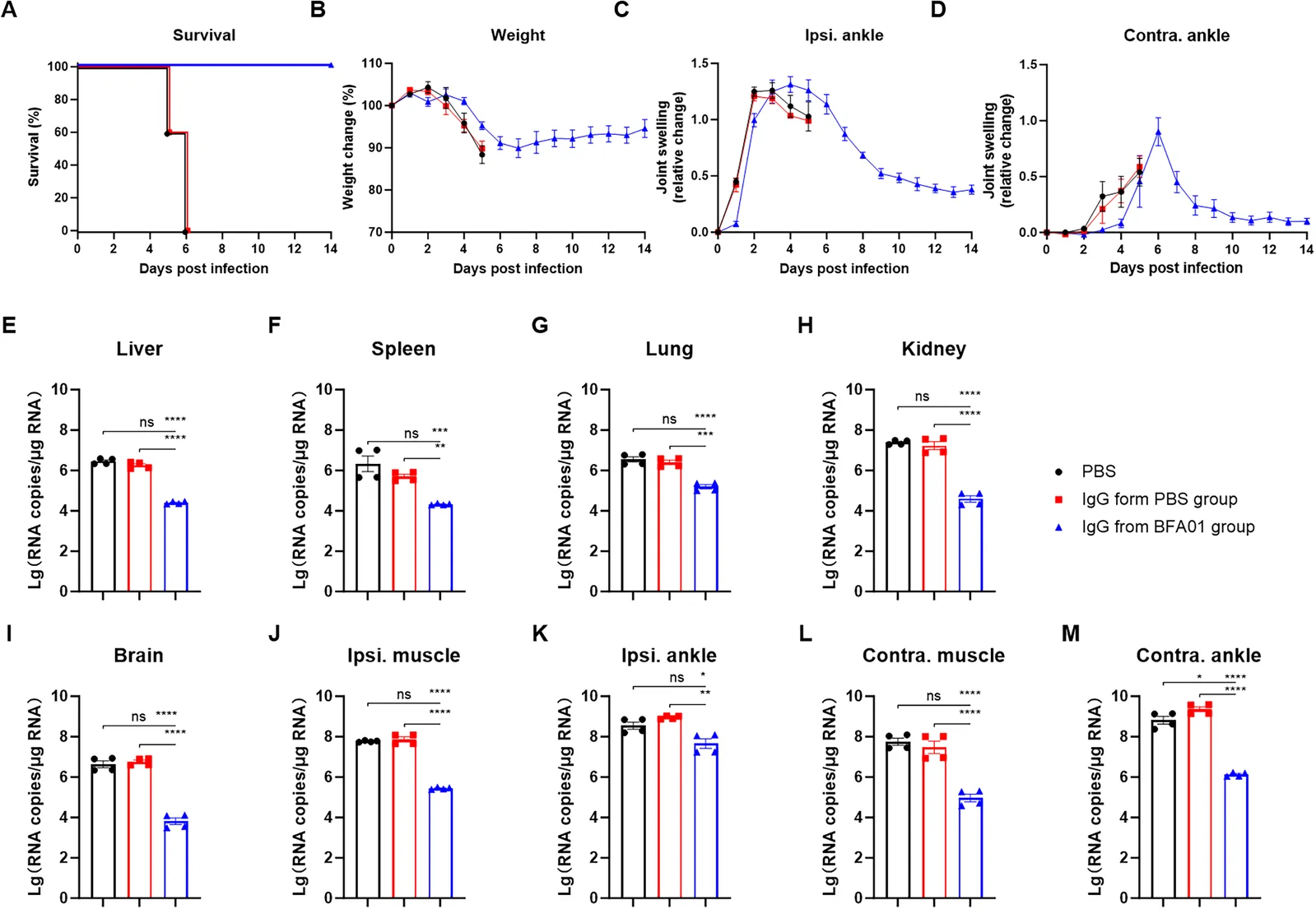
Protection of purified IgG via passive transfer. PBS or IgG purified from different groups were transferred into naive A129 mice. 24 hours later, the mice were challenged with attenuated CHIKV. Survival **(A)**, weight change **(B)**, ipsilateral joint swelling **(C)**, and contralateral joint swelling **(D)** were monitored 14 days after infection. Data are represented as mean ± SEM. *n* = 5/group (5 females). Virological assessment by RT-qPCR at 3 dpi in the liver **(E)**, spleen **(F)**, lung **(G)**, kidney **(H)**, brain **(I)**, ipsilateral ankle **(J)**/muscle **(K)**, and contralateral ankle **(L)**/muscle **(M)**. Significance was determined by one-way analysis of variance with Tukey’s multiple-comparison test; *p<0.05, **p<0.01, ***p<0.001, ****p<0.0001; ns, not significant. Data are represented as mean ± SEM. *n* = 2/group (two females).

Viral loads in tissues were similarly determined on day 3 post-infection. IgG from mice immunized with BFA01-adjuvanted vaccine significantly reduced viral loads in most tissues (**Figures 7E–M**). These results indicate that the humoral response elicited by this subunit vaccine is sufficient to protect mice from lethal challenge.

## Discussion

CHIKV epidemics are explosive, rapidly moving, and unpredictable (44). The rising incidence of CHIKV infections in tropical and subtropical regions poses a growing threat to public health and quality of life. CHIKV infection can lead to significant disability and imposes a substantial economic burden in endemic regions. Therefore, safe and effective vaccines are urgently needed to prevent infection and chronic disease associated with CHIKV worldwide.

In this study, we developed a CHIKV subunit protein vaccine and evaluated its immunological and protective efficacy in mice. The results showed that two doses of all three formulations effectively protected A129 mice from lethal CHIKV challenge. Notably, as little as 1 μg subunit protein adjuvanted with BFA01 not only protected mice from death but also prevented joint swelling. In addition, a single dose of 5 μg subunit protein adjuvanted with BFA01 also protected mice from death and significantly alleviated joint swelling.

It is believed that mouse Th2 cells enhance IgG1 synthesis, while Th1 cells promote IgG2a production (45). Thus, IgG1 and IgG2a levels reflect, to some degree, Th2- and Th1-biased immune responses, respectively. In our study, the aluminum-adjuvanted vaccine elicited high levels of IgG1 and low levels of IgG2a in A129 mice, suggesting a Th2-biased immune response. In contrast, The BFA01-adjuvanted vaccines elicited high levels of both IgG1 and IgG2a, suggesting a relatively balanced Th1/Th2 immune response. In humans, CHIKV-neutralizing antibodies are mainly IgG3 (43). In our study, the BFA01-adjuvanted vaccines elicited significantly higher levels of IgG3 in A129 mice than the aluminum-adjuvanted vaccine, which was consistent with the observed degree of joint swelling. The IgG3 levels seemed to be constituent with the protection efficacy; however, whether a direct correlation exists between them remains to be further investigated.

Currently, only two vaccines have been licensed in certain regions. The marketing of the live-attenuated CHIKV vaccine was recently suspended in the USA due to severe adverse events (33). It is not suitable for pregnant women or individuals who are immunodeficient or immunosuppressed due to disease, condition, or medical therapy. Individuals aged over 65 years with one or more chronic medical conditions may also have an increased risk of severe adverse events (46). Moreover, the vaccine is freeze-thaw sensitive and costive, estimated at $350 per dose (36). The other licensed vaccine, a virus-like particle vaccine, demonstrated a favorable safety profile and high immunogenicity (24, 34, 47). However, the manufacturing process is technically demanding, which may constrain large-scale production and drive up costs (35). Safety is the most important characteristic of subunit vaccines, as they do not involve infectious viruses (48) and their production does not require high level biosafety laboratories. In addition, they are less likely to induce side effects at the injection site (49). Therefore, subunit vaccines are highly suitable for production and use in low- and middle-income countries.

Originally, three major genetic lineages of CHIKV were defined: West African, Asian, and East/Central/South African (ECSA) (50). Subsequently, a fourth lineage, the Indian Ocean Lineage (IOL), was identified as a direct descendant of the ECSA lineage (50, 51). CHIKV is believed to exist as a single serotype (51, 52), enabling cross-protection against all lineages (34). Consistent with this, virus-like particles (VLPs) derived from ancestral CHIKV strains (37997 and S27) have been reported to confer robust protection against recent outbreak strains belonging to the Indian Ocean Lineage (53, 54). Similarly, our subunit vaccine, based on the strain 37997 sequence, also conferred robust protection against attenuated LR2006 strain (IOL). Our data revealed that the recombinant protein based on the strain 37997 sequence elicited high-titer cross-reactive antibodies and potent pseudotyped virus neutralization activity against heterologous lineages, suggesting the vaccine’s cross-protective potential; however, this remains to be validated using authentic wild-type virus.

Epitopes targeted by neutralizing monoclonal antibodies from infected individuals map to both E1 and E2 glycoproteins, indicating that both envelope glycoproteins can elicit neutralizing responses. These antibodies inhibit viral infection through diverse mechanisms, including blocking receptor binding, preventing viral entry, interfering with membrane fusion, and impeding virion release. Epitopes targeted by neutralizing monoclonal antibodies from infected individuals map to both E1 and E2 glycoproteins (40, 55), indicating that both envelope glycoproteins can elicit neutralizing responses. These antibodies inhibit viral infection through diverse mechanisms, including blocking receptor binding, preventing viral entry, interfering with membrane fusion, and impeding virion release. Therefore, both E1 and E2 are frequently selected as immunogens across diverse vaccine platforms. As the E2 envelope glycoprotein is more surface-exposed than E1, neutralizing antibodies preferentially target epitopes within E2 (56, 57). By contrast, E1 exhibits a lower degree of amino acid variation (58, 59) and contains more conserved cross-reactive epitopes than E2 (60). Because of its structural and conformational instability, E2 exhibits insufficient immunogenicity when expressed alone. Previous studies have shown that recombinant E1 and E2 proteins often elicit only low antibody responses (61); however, combining E1 and E2 into a cocktail induces higher levels of neutralizing antibodies than either protein alone (30). Therefore, antigen design should involve either co-expression of all structural proteins or, at a minimum, inclusion of the E3-E2-E1 polyprotein (33). Crystallographic analysis of the E2-E1 complex expressed in S2 cells revealed a heterodimeric structure closely resembling that observed in native virion spike complexes (16). Following this rationale, we designed the sequence of the recombinant protein.

Mammalian expression systems have been increasingly explored for vaccine production. As natural hosts for CHIKV replication, mammalian cells perform authentic post-translational modifications (62, 63), including complex N-glycosylation and accurate disulfide bond formation, which are essential for proper folding of the envelope glycoproteins. While numerous studies have assessed CHIKV envelope protein immunogenicity, many have relied on bacterial or insect cell expression systems (30, 42, 61, 64–69). In bacterial hosts, the lack of proper folding frequently leads to inclusion body formation, and subsequent renaturation rarely achieves complete restoration of native conformational epitopes. Structural studies of the CHIKV p62-E1 heterodimer have identified functionally important glycosylation sites at E1-Asn141 and E2-Asn263. Comparative analyses have further demonstrated that virus-like particles produced in HEK293 cells exhibit distinct N-glycan profiles compared to those from Sf9 insect cells. In this context, the robust neutralizing antibody responses observed in the present study support the premise that mammalian cell-derived antigens preserve immunologically relevant conformations that might be compromised in alternative expression platforms.

Adjuvants serve as critical components of vaccine formulations, amplifying the immunogenicity of target antigens and substantially improving protective outcomes. As the most widely used adjuvants in human vaccines, aluminum-based adjuvants have been clinically approved for decades. Aluminum adjuvants bind antigens through electrostatic interactions, forming particulate complexes that facilitate delivery to antigen-presenting cells (APCs) and enhance cellular uptake. However, aluminum adjuvants have limitations in effectively inducing robust cellular immune responses and may lead to adverse reactions, primarily due to its low immunogenicity and irritability (70). This limitation has driven the development of novel adjuvants. BFA01 is a liposomal adjuvant composed of monophosphoryl lipid A (71) and the saponin QS-21. MPL activates the innate immune system via TLR4 binding, primarily through a TRIF-dependent signaling pathway (72). Mouse model studies have demonstrated that QS-21 activates caspase-1 in subcapsular sinus macrophages (SSMs) within draining lymph nodes, thereby playing a critical role in immune response regulation (73). BFA03 contains squalane and α-tocopherol; the latter acts as an immunostimulant that enhances secretion of chemokines and cytokines (e.g., CCL2, CCL3, IL-6, and CXCL1), promotes antigen uptake by APCs, and increases granulocyte recruitment to draining lymph nodes (74). BFA04 comprises MPL adsorbed onto an aluminum salt adjuvant, thereby combining the immunostimulatory properties of MPL with the depot effect of aluminum. Our data show that BFA01 adjuvant promotes a relatively balanced Th1/Th2 response and effectively reduces the required antigen dose, protecting mice from lethal challenge even with 1 μg of subunit protein.

While the present study establishes the immunogenicity and protective efficacy of mammalian cell-derived subunit vaccine in a murine model, several limitations warrant consideration and point toward future investigations. First, all *in vivo* assessments were conducted in mice; translation to non-human primates, which more closely recapitulate human CHIKV pathogenesis and immune responses, remains to be established. Second, protective efficacy was demonstrated only against the attenuated CHIKV LR2006 strain. Attenuated strains cannot fully mirror the viremia magnitude or pathogenic cascade of wild-type CHIKV infection. Although cross-lineage binding and pseudotyped virus neutralization data provide preliminary support for broad reactivity, pseudotyped systems cannot replicate the complete biology of authentic virions. Definitive validation using virulent wild-type isolates from multiple lineages is therefore essential. Such experiments require BSL-3 facilities and institutional approvals that we currently lack; we are actively pursuing partnerships with BSL-3-equipped laboratories to complete these critical studies. Third, the present study focused primarily on humoral immune endpoints. Cellular immune responses were assessed only in a small pilot cohort, and future studies will employ larger sample sizes and multiparameter T-cell profiling to fully characterize the cellular immunogenicity of these vaccine formulations. Fourth, the present study did not evaluate long-term protective immunity conferred by the BFA01-adjuvanted formulation. While preliminary observations from our previous studies revealed that the aluminum-adjuvanted formulation elicited sustained antibody responses (**Supplementary Figure S3**), the durability of neutralizing titers and immunological memory specifically for the BFA01 adjuvanted formulation remain to be directly assessed. Future longitudinal studies will carefully evaluate the maintenance of antibody levels and memory B-cell responses over an extended period.

In summary, our data demonstrated that CHIKV subunit protein adjuvanted with suitable adjuvants exhibits good immunogenicity in mice. Two doses of immunization protected A129 mice from death and joint swelling, and significantly reduced viral loads. A single dose also afforded partial protection against mortality, alleviated weight loss, and relieved joint swelling. Among the adjuvants tested, BFA01 was the most effective. These results suggest that the subunit adjuvanted with BFA01 may be a highly promising vaccine candidate against CHIKV.

## Data Availability

The raw data supporting the conclusions of this article will be made available by the authors, without undue reservation.
