# Supplementary material for: A subunit vaccine protects A129 mice from death and joint swelling against lethal Chikungunya virus challenge
Source: Front Immunol. 2026 Oct 6;17:1937609. doi: 10.3389/fimmu.2026.1937609 (PMC13680182; doi:10.3389/fimmu.2026.1937609)
Supplement: Supplementary file 1 [file DataSheet1.docx]

**Supplementary figures**

**Figure S1.** IFN-γ ELISpot. ELISpot was performed one week post boost. SFU: spot forming units. Data are represented as mean ± SEM. *n* = 2 (one female, one male).

**Figure S2.** Histological analysis of tissues from two-dose-vaccinated mice on day 3 post-infection. All images have the same magnification. Scale bar, 200 µm.

**Figure S3.** Long-term surveillance of IgG elicited by aluminum-adjuvanted subunit vaccine. Data are represented as mean ± SEM. n = 5/group (5 females).
